# An Unusual Case of Uremic Tumoral Calcinosis with Atypical Manifestation in a Patient on Peritoneal Dialysis: Case Report and Review of the Literature

**DOI:** 10.3390/medsci13010011

**Published:** 2025-01-29

**Authors:** Esperanza Moral Berrio, Roger A. Cox Conforme, Raúl Elías, José C. De La Flor, Celia Rodríguez Tudero, María Dolores Sánchez de la Nieta-García, Rocío Zamora González-Mariño, Carmen Vozmediano Poyatos

**Affiliations:** 1 Department of Nephrology, Hospital General Universitario de Ciudad Real, 13005 Ciudad Real, Spain; emoral@sescam.jccm.es (E.M.B.); rcox@sescam.jccm.es (R.A.C.C.); mdoloressd@sescam.jccm.es (M.D.S.d.l.N.-G.); mcarmenv@sescam.jccm.es (C.V.P.); 2 Department of Nephrology, Hospital Cayetano Heredia, Lima 15002, Peru; raul.elias.c@upch.pe; 3 Department of Nephrology, Hospital Central Defense Gomez Ulla, 280467 Madrid, Spain; 4 Faculty of Medicine, Alcala de Henares University, 28805 Madrid, Spain; 5 Health Sciences Doctoral Program, Faculty of Medicine, Alcala University, 28805 Madrid, Spain; 6 Department of Nephrology, Hospital Universitario de Salamanca, 37007 Salamanca, Spain; crodrigueztudero@usal.es; 7 PhD in Surgery Department, Faculty of Medicine, University of Salamanca, 37007 Salamanca, Spain; 8 Department of Nephrology, Hospital Universitario General Villalba, 28400 Madrid, Spain; rocio.zamora@quironsalud.es

**Keywords:** uremic tumoral calcinosis, end-stage renal disease, peritoneal dialysis, hyperphosphatemia, chronic kidney disease mineral and bone disorder

## Abstract

Background: Uremic tumoral calcinosis (UTC) is a rare yet severe complication of chronic kidney disease (CKD), predominantly occurring in patients undergoing renal replacement therapy (RRT). It is characterized by extensive soft tissue calcifications, frequently associated with chronic hyperphosphatemia and disruptions to calcium–phosphorus metabolism. Case report: This report describes a 34-year-old woman with end-stage renal disease (ESRD) secondary to lupus nephritis, undergoing continuous ambulatory peritoneal dialysis (CAPD). She presented with a progressively enlarging calcified mass in the proximal phalanx of the third finger on her right hand, accompanied by functional impairment. Laboratory findings revealed persistent hyperphosphatemia (8.8 mg/dL), elevated parathyroid hormone levels (901 pg/mL), and low vitamin D levels (9 ng/mL), indicating significant disturbances to mineral metabolism. Imaging studies, including X-ray and whole-body 18F-Choline positron emission tomography/computed tomography (PET/CT), confirmed the presence of localized calcifications in the soft tissue of the proximal phalanx of the third finger on her right hand and parathyroid hyperplasia, respectively. Initial management included the optimization of phosphate binders and calcimimetic therapy, with the subsequent intensification of dialysis therapy. Transitioning to automated peritoneal dialysis (APD) with high-volume exchanges resulted in a notable improvement in biochemical parameters and the eventual remission of the calcified mass. Conclusion: This case underscores the importance of comprehensive management in dialysis patients, including dietary phosphate restriction, the appropriate use of non-calcium-based binders, and tailored dialysis regimens to prevent and treat CKD-related mineral and bone disorders. It also highlights the utility of imaging modalities such as PET/CT in diagnosing UTC and monitoring response to therapy. Further research is needed to elucidate the pathophysiology of UTC and optimize its management in dialysis patients.

## 1. Introduction

Uremic tumoral calcinosis (UTC) is a rare manifestation of chronic kidney disease (CKD), characterized by abnormal calcium and phosphate deposits in soft tissues. These deposits typically present as pseudo-tumoral masses, predominantly in periarticular areas and subcutaneous tissues [1]. While its occurrence is strongly associated with alterations in CKD–mineral bone disease (MBD)—particularly hyperphosphatemia and impaired calcium handling leading to the precipitation of calcium-phosphate crystals in soft tissues—UTC remains a poorly understood clinical phenomenon [2]. This condition is most frequently observed in patients with end-stage renal disease (ESRD) undergoing dialysis treatment, including both hemodialysis (HD) and peritoneal dialysis (PD) [3,4].

In patients on PD, although renal replacement therapy (RRT) provides some capacity for phosphate removal, its efficacy is often insufficient to maintain phosphate levels within the normal range [5]. This imbalance is further exacerbated by dietary phosphate intake, declining residual kidney function (RKF), and variable adherence to phosphate binders. Persistent hyperphosphatemia is therefore a major risk factor in the development of UTC in these patients. UTC clinically manifests as slow-growing calcified masses in periarticular areas, but it can also affect subcutaneous tissues, tendons, and ligaments. While typically painless, these masses may occasionally cause discomfort by compressing adjacent structures or limiting joint mobility. Diagnosis is based on a combination of clinical findings, biochemical analysis, and imaging studies. Patients often present with chronic hyperphosphatemia, elevated parathyroid hormone (PTH) levels, and, in some cases, hypocalcemia. Imaging modalities such as X-rays and computed tomography (CT) are useful in assessing the extent of calcifications in soft tissues, while magnetic resonance imaging (MRI) can provide detailed a visualization of the affected structures and help rule out alternative pathologies [6].

PD offers several advantages for patients with ESRD, including flexibility, more continuous fluid and solute removal, and less reliance on permanent vascular access. However, one of its primary limitations compared to HD is its relatively lower efficacy in phosphate clearance [7]. The peritoneal membrane facilitates the removal of small solutes, but phosphate, primarily located in the intracellular space, requires more effective mechanisms for its elimination. As CKD progresses and RKF diminishes, phosphate clearance becomes increasingly compromised. Without adequate dietary phosphate restriction or optimal use of phosphate binders, PD alone may not suffice to control hyperphosphatemia, directly contributing to CKD-MBD complications, including UTC [5].

The management of UTC in dialysis patients requires a comprehensive approach that includes dietary phosphate restriction, the use of non-calcium-based phosphate binders (such as sevelamer or lanthanum carbonate), and the optimization of dialysis regimens. In certain cases, the intensification of the PD protocol or transitioning to HD may be necessary to achieve better phosphate control. Additionally, complications such as secondary hyperparathyroidism (SHPTH) should be addressed using calcimimetics or, in refractory cases, parathyroidectomy [5].

In this report, we present the case of a PD patient who developed UTC in the proximal phalanx of the third finger on her right hand. We detail her clinical presentation, the adjustments made to her medical management and dialysis regimen that resulted in complete remission, and the challenges associated with managing this rare complication in patients with ESRD on PD.

## 2. Case Presentation

We report the case of a 34-year-old woman with ESRD secondary to lupus nephritis, who had been undergoing renal RRT with continuous ambulatory peritoneal dialysis (CAPD) since March 2022. Her dialysis regimen consisted of one bag of 2 L (L) of Physioneal 40 (calcium (Ca) 1.25 mM) 1.36%, two bags of 2 L of Physioneal 35 (Ca 1.75 mM) 2.27%, and one bag of 2 L of Extraneal. Based on the dialysate-to-plasma ratio of creatinine (D/P Cr) of 0.72, her peritoneal membrane was classified as having an average–high transport type. Residual diuresis was approximately 900 mL/day, with a weekly Kt/V of 2.14 and a weekly creatinine clearance (CCr) of 58.14 L. Her treatment for CKD-MBD and SHPTH included 30 mg cinacalcet daily, 9.6 g sevelamer daily divided into three doses, and 0.266 mg calcifediol monthly.

**Figure 1:**
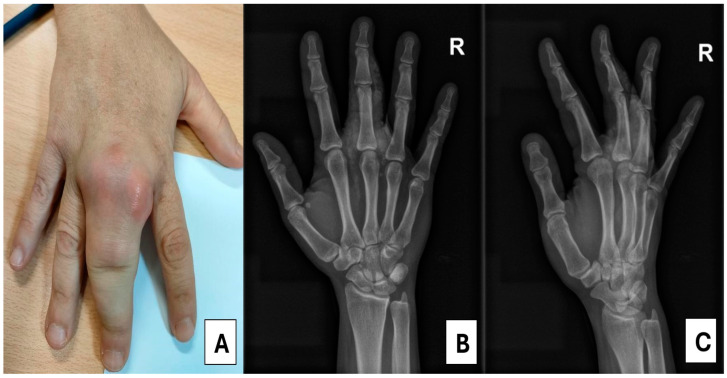
(**A**) Right hand showing a lobulated, cystic-appearing mass; (**B**,**C**) X-rays of the right hand revealing calcifications localized in the proximal phalanx of the third finger.

**Figure 2:**
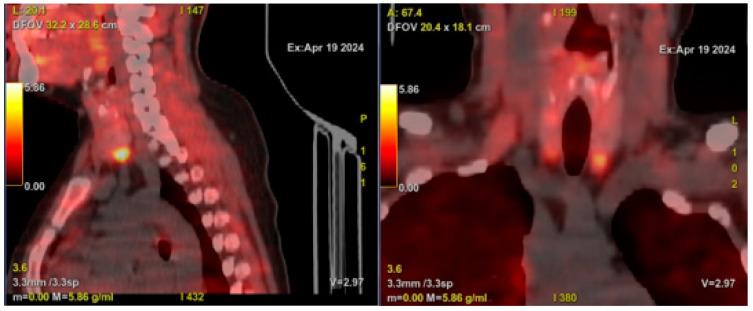
Whole-body 18F-Choline positron emission tomography/computed tomography (PET/CT) demonstrating hyperplasia of all four parathyroid glands, with the inferior glands, particularly the left one, exhibiting prominent size and metabolic activity.

In November 2023, the patient presented with progressive swelling of the proximal phalanx of the third finger on her right hand, accompanied by functional limitation (Figure 1A). A plain X-ray of the affected hand revealed a well-defined, calcified, periarticular lobulated mass, with no involvement of the bone cortex in the proximal phalanx (Figure 1B,C). Laboratory tests showed a serum Ca (sCa) level of 9.9 mg/dL (normal range [NR]: 8.6–10.2 mg/dL), serum phosphorus (sP) of 8.8 mg/dL (NR: 2.5–4.5 mg/dL), serum alkaline phosphatase of 68 IU/L (NR: 39–105 IU/L), parathyroid hormone (PTH) of 901 pg/mL (NR: 15–65 pg/mL), and 25-hydroxy vitamin D of 9 ng/mL (NR: >30 ng/mL) (Table 1). Whole-body 18F-Choline positron emission tomography/computed tomography (PET/CT) identified hyperplasia of all four parathyroid glands, with the inferior glands, particularly the left one, showing increased size and metabolism (Figure 2).

The initial treatment adjustments included increasing the oral calcimimetic dose to cinacalcet 60 mg five days per week and 30 mg two days per week, while maintaining sevelamer at 9.6 g daily, which resulted in a minimal response. Subsequently, the patient was transitioned to automated peritoneal dialysis (APD) with a regimen consisting of one bag of 5 L of Physioneal 40 (Ca 1.25 mM) 1.36%, one bag of 5 L of Physioneal 40 (Ca 1.25 mM) 2.27%, one bag of 2.5 L of Nutrineal, and a final infusion of 2 L of Extraneal. Additionally, the cinacalcet dose was further increased to 90 mg four days per week and 120 mg three days per week. A left humeral–cephalic arteriovenous fistula was created in preparation for potential HD initiation if remission of UTC symptoms did not occur; the fistula was functional during follow-up.

**Figure 3:**
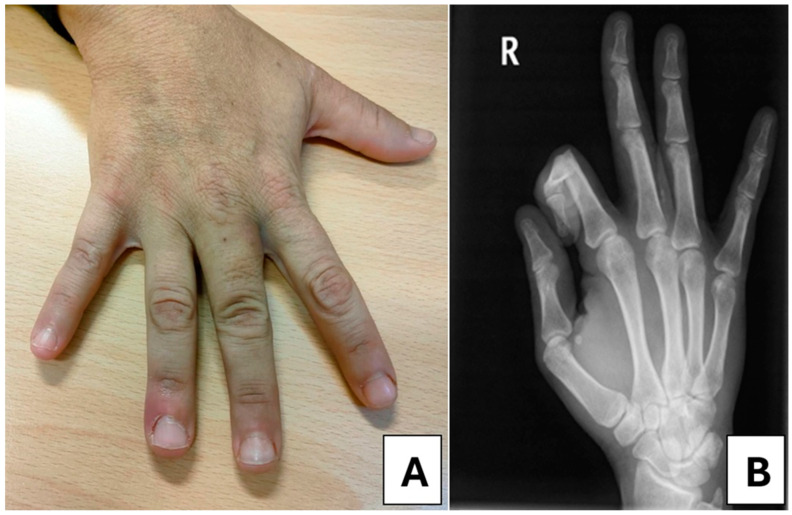
(**A**) Photograph of the right hand demonstrating the resolution of the calcified mass after treatment; (**B**) X-ray of the right hand showing the absence of calcifications and the full resolution of the condition.

With close monitoring, the mass showed near-complete remission and eventually disappeared (Figure 3A,B), accompanied by a progressive decrease in PTH and sP levels, as well as improved sCa control. These changes resulted in significant clinical improvement for the patient.

## 3. Discussion

We present the case of a young woman on PD who developed a tumoral mass in the metacarpophalangeal joint of her right hand, which limited its functionality. Imaging and laboratory studies confirmed the diagnosis of UTC. This is one of the few reported cases of UTC to have achieved remission with medical management alone.

UTC is a rare complication observed in patients with ESRD. Its incidence in PD patients is approximately 1.6%, similar to that reported in HD patients, as noted in the retrospective cohort study by Chu HY et al. [5]. The onset of UTC in PD patients typically occurs 24–48 months after initiating therapy, according to the literature (Table 2). However, cases with shorter durations, such as those reported by Fatehi M et al. [8] and Maioli ME et al. [9], where UTC manifested within 2 and 6 months, respectively, were preceded by other forms of RRT before starting PD. This highlights the role of prolonged CKD-MBD alterations, including a persistently high calcium–phosphorus product (Ca × P) and uncontrolled SHPTH, in the pathophysiology of UTC. Proposed mechanisms include repeated trauma, necrosis, fibrosis, and calcification pathways [10]. In our patient, the relatively short duration of PD before UTC onset suggests that pre-existing CKD-MBD alterations predisposed her to the development of extraosseous calcifications.

Most UTC lesions are multifocal and predominantly affect overused joints, such as the shoulders, elbows, hips, and knees [5]. However, in our case, only a single joint of the hand was affected. This atypical presentation could be attributed to the short disease duration and the timely and effective management instituted. Other unusual locations, such as the metatarsophalangeal joint, have been reported, particularly in female patients, potentially associated with factors like wearing high-heeled shoes [11]. In our case, the location was even more uncommon and could not be explained by mechanical or traumatic factors.

Our patient presented with elevated Ca × P levels and significantly high PTH levels. A Ca × P above 60 mg^2^/dL^2^ and PTH levels exceeding 400 pg/mL are known to facilitate extraosseous calcification [3,5,6,7,8,9,10]. Interestingly, cases of UTC in HD patients have been reported where PTH levels were not excessively elevated, but calcitriol levels were high despite low 25-hydroxy vitamin D levels, suggesting endogenous calcitriol production by granulomas [12]. In such cases, assessing calcitriol levels may help refine diagnosis. However, this was not necessary in our patient, given the evident Ca × P alterations and hyperparathyroidism. Other predisposing factors for UTC include hypoparathyroidism, adynamic bone disease, aluminum toxicity, the excessive use of calcium-based phosphate binders, and alkalemia [5], none of which were present in our case.

UTC diagnosis is primarily one of exclusion. Imaging plays a critical role, with magnetic resonance imaging (MRI) revealing two distinct patterns: (a) a diffuse pattern with low signal intensity and (b) a nodular pattern with alternating high-signal-intensity areas and signal voids [2]. MRI can also delineate the extent of calcifications and their relationship with surrounding structures, aiding in distinguishing UTC from other conditions [2].

The medical management of UTC includes dietary phosphorus restriction, non-calcium phosphate binders, adjustments to dialysis therapy, low-calcium dialysis solutions, and calcimimetics [5]. Among the 26 cases reviewed in the literature, only two cases, reported by Kim Y et al. [12] and Raju DL et al. [13], achieved complete remission with medical therapy. In both cases, the patients remained on peritoneal dialysis. The patient in the case report by Kim Y et al. [12] discontinued calcium-based phosphate binders and vitamin D, initiated non-calcium phosphate binders, and used low-calcium dialysis solutions. Vitamin D was reintroduced 12 months later, and remission was achieved after 3 years (Table 2). Unlike our case, calcimimetics were not used, and the lesions were more severe, affecting heavily loaded joints like the shoulders and hips. This suggests that the addition of calcimimetics, combined with early recognition and management, may benefit certain patients [14]. However, González et al. [10] reported a case of severe UTC where relapse occurred despite calcimimetic use.

In our patient, parathyroidectomy was considered but deferred due to the excellent response to medical management. This decision is supported by the literature, indicating a significant recurrence rate (up to 66%) following surgical treatment [3,5,15]. Aristizabal-Alzate A et al. [16] described a case where parathyroidectomy induced complete remission, particularly in tertiary hyperparathyroidism. For our patient, medical management proved effective, but parathyroidectomy remains a potential option in the event of recurrence, especially given the PET/CT findings suggesting parathyroid hyperplasia.

In up to 8 of the 26 cases described [8,9,17,18,19,20,21,22], patients switched to hemodialysis, achieving complete remission, since phosphate is much more effectively cleared by HD than by PD. In our case, creating a functional arteriovenous fistula was prioritized to prepare for this possibility.

Other cases of UTC have required surgical interventions, including local lesion resection [15,23,24], parathyroidectomy [5,16,17,18,21,25], or renal transplantation [5], to achieve resolution. The remaining case reports are shown in Table 2 [26,27,28]. 

**Table 2:** **Previous cases of uremic tumoral calcinosis in peritoneal dialysis patients**. APD, ambulatory peritoneal dialysis; B, bilateral; CAPD, continuous ambulatory peritoneal dialysis; Ca, calcium; CCr, creatinine clearance; HD, hemodialysis; IP, interphalangeal joint; MCP, metacarpophalangeal joint; MTP, metatarsophalangeal joint; P, phosphorus; PD, peritoneal dialysis; PET, peritoneal equilibration test; PTH, parathyroid hormone.

	Chang CC. et al. (2013) [23]	Kim Y. et al. (2014) [12]	González G. et al. (2015) [10]	Maioli ME. et al. (2017) [9]	Zhou H. et al. (2018) [25]	Chou YP. et al. (2022) [15]	Jayanatha K. et al. (2024) [17]
Age (years)	44	25	46	22	37	55	28
Sex	Female	Male	Male	Male	Female	Female	Female
Duration of PD (months)	120	108	60	6	28	108	28
Modality	CAPD	CAPD	APD	CAPD	CAPD	-	-
Weekly Kt/V	1.82	1.633	1.65	-	1.95	1.77	1.695
Weekly CCr (L)	62	-	-	-	-	53.48	40.87
Clinical presentation	Periodontoid region (atlantoaxial) Elbow (B) Wrist	Shoulder (B) Hip (B) Gluteus (B)	Shoulder Hip (B) Gluteus (B) Thigh (B)	Hand (MCP, IP) Arm	Shoulder (B) Elbow Wrist	Back	Elbow (B) Wrist (B) Hand (MCP, IP) (B)
PTH (pg/mL)	417	417	185–607	1867	61.8–134.3	1176	990
Ca (mg/dL)	8.0	9.9	9–10	8.5	9.6–10.8	10.5	10.42
P (mg/dL)	9.9	3.7	4.6–6	11.1	4.0–7.1	8.1	7.68
Ca × P (mg^2^/dL^2^)	79.2	36.63	41.4–60	94.35	38.4–76.6	85.05	80
Treatment	Surgical excision followed by medical treatment	Medical treatment	Medical treatment, surgical excision	Medical treatment, transfer to HD	Medical treatment followed by parathyroidectomy	Medical treatment followed by surgical excision	Transfer to HD, parathyroidec-tomy
Outcome	Partial remission	Complete remission	Resistance	Complete remission	Complete remission after parathyroidectomy	Remission after surgery	Complete remission
	Aristizabal-Alzate A. et al. (2022) [16]	Holub T. et al. (2022) [18]	Fatehi M. et al. (2016) [8]	Kuriyama S. et al. (1998) [19]	Raju D. L. et al. (2006) [13]	Guo R. et al. (2017) [24]	Floege J. (2004) [26]
Age (years)	48	28	73	32	39	55	53
Sex	Female	Male	Female	Male	Male	Female	Male
Duration of PD (months)	36	42	2	24	48	60	12
Modality	-	-	CAPD	CAPD	CAPD	-	-
Weekly Kt/V	-	-	-	-	-	-	-
Weekly CCr (L)	-	-	-	-	-	-	-
Clinical presentation	Hand (IP) (B) Shoulder	Shoulder (B)	Paraspinal soft tissues adjacent to the left C2–3 facet joint	Hand (B) Elbow (B) Knee (B)	Left mandibular gland Hand (IP) Shoulder	Right intervertebral foramens at C4-C5 and C5-C6 Anterior wall of the spinal canal	Skull Conjunctiva Sternum Lungs Thyroid glandHand (IP)
PTH (pg/mL)	2362	1314.5	-	42	591	-	-
Ca (mg/dL)	10.2	9.86	-	10.42	8.72	-	14.87
P (mg/dL)	9.7	9.11	8.27	6.2	10.6	-	-
Ca × P (mg^2^/dL^2^)	98.94	89.82	78.92	64.6	92.4	-	-
Treatment	Parathyroidectomy	Medical treatment, transfer to HD, and parathyroidectomy	Transfer to HD	Medical treatment and combined therapy with HD and CAPD with low-Ca dialysate	Medical treatment and CAPD	Surgical excision	-
Outcome	Partial remission	Partial remission	Progressive improvement	Complete remission	Complete remission	Complete remission	-
	Al-ani M. et al. (2016) [20]	Van Straten A. et al. (2005) [21]	Chu H. et al. (2011) [5]	Chu H. et al. (2011) [5]	Chu H. et al. (2011) [5]	Chu H. et al. (2011) [5]	Chu H. et al. (2011) [5]
Age (years)	22	30	54	38	35	21	55
Sex	Female	Male	Male	Female	Female	Female	Female
Duration of PD (months)	24	60	72	84	63	17	32
Modality	-	CAPD	CAPD	CAPD	CAPD	CAPD	CAPD
Weekly Kt/V	-	-	2.59	2.01	5.51	1.84	2.05
Weekly CCr (L)	-	-	80.9	63.3	61.11	47.82	50.05
Clinical presentation	Sternoclavicular joint (B)	Sternoclavicular joint Distal end of the left clavicle	Shoulder (B) Hand (MCP) Hip (B) Foot (MTP)	Shoulder Wrist Hand (MCP) Hip Ankle	Hip Shoulder	Hand (MCP) (B) Foot (IP) (B)	Shoulder (B)
PTH (pg/mL)	2308.7	>2499	519	676	242	756	700
Ca (mg/dL)	10.4	-	10.1	10.7	9.8	8.8	10.5
P (mg/dL)	9.7	>12.4	7.1	7.3	7.9	8.9	8.5
Ca × P (mg^2^/dL^2^)	101	>124	71.1	78.1	77.4	78.3	89.3
Treatment	Medical treatment and transfer to HD	Medical treatment, transfer to HD, and parathyroidectomy	Medical treatment, parathyroidectomy, and renal transplantation	Medical treatment, parathyroidectomy, and renal transplantation	Medical treatment	Medical treatment and renal transplantation	Medical treatment
Outcome	-	Complete remission	Complete remission	Complete remission	Resistance	Complete remission	Resistance
	Chu H. et al. (2011) [5]	Chu H. et al. (2011) [5]	Shpilberg K A. et al. (2013) [27]	Kim J. et al. (2023) [28]	Kamar F B. et al. (2016) [22,28]		
Age (years)	51	42	42	71	54		
Sex	Female	Female	Female	Female	Female		
Duration of PD (months)	23	26	8	36	-		
Modality	CAPD	APD	-	-	APD		
Weekly Kt/V	1.84	1.78	-	-	2.72		
Weekly CCr (L)	47.82	51.1	-	-	-		
Clinical presentation	Shoulder Elbow (B) Wrist (B) Hand (MCP) (B) Foot (MTP) (B)	Shoulder Hip	Neck Shoulder Elbow Back Hip Foot	Neck	Cervical spine Shoulder (B) Sternoclavicular joint Hip (B) Gluteus Foot		
PTH (pg/mL)	168	1085	780	763.6	332		
Ca (mg/dL)	9.9	11.8	9.3	6.4	11		
P (mg/dL)	7	8.7	8	13.4	6.3		
Ca × P (mg^2^/dL^2^)	69.3	102.7	74.4	85.76	69		
Treatment	Medical treatment and renal transplantation	Medical treatment and parathyroidectomy	Medical treatment Pending parathyroidectomy	Medical treatment	Transfer to HD		
Outcome	Complete remission	Partial remission	Resistance to medical treatment	-	Complete remission		

## 4. Conclusions

This case underscores the complexity of managing UTC in patients with ESRD on PD. It demonstrates how severe disturbances in CKD-MBD, including persistent hyperphosphatemia and SHPTH, can lead to rare yet debilitating complications. The transition from continuous ambulatory peritoneal dialysis (CAPD) to an intensive automated peritoneal dialysis (APD) regimen, combined with the optimization of calcimimetics and non-calcium phosphate binders, not only achieved control of metabolic parameters but also led to the resolution of calcified lesions and a significant improvement in the patient’s quality of life.

This case highlights the importance of adopting a comprehensive and dynamic approach to dialysis therapy and the critical role of advanced imaging modalities, such as PET-CT, in the accurate diagnosis and follow-up of UTC. Further research is essential in better understanding the underlying pathological mechanisms and developing more effective therapeutic strategies for preventing and treating this condition in patients with ESRD.

## Figures and Tables

**Table 1:** Characteristics of peritoneal dialysis, therapy, and laboratory tests.

	November 2024	January 2024	March 2024	May 2024	June 2024
Modality	CAPD	CAPD	ADP	ADP	ADP
PET category	Average high transport	----	----	-----	High transport
D/P Cr	0.72	----	-----	----	0.92
Weekly Kt/V	2.14	-----	----	2.99	---
Weekly CCr (L)	58.14	----	----	85.83	----
Residual diuresis (mL/24 h)	625	900	900	1000	700
Peritoneal dialysis schedule	2 L of Physioneal 40 Glucose 1.36% 2 L of Physioneal 35 Glucose 2.27% 2 L of Physioneal 35 Glucose 2.27% 2 L of Extraneal	2 L of Physioneal 40 Glucose 1.36% 2 L of Physioneal 40 Glucose 2.27% 2 L of Physioneal 40 Glucose 2.27% 2 L of Extraneal	5 L of Physioneal 40 Glucose 1.36% 5 L of Physioneal 40 Glucose 2.27% 2.5 L of Nutrineal 2.5 L of Extraneal	5 L of Physioneal 40 Glucose 1.36% 5 L of Physioneal 40 Glucose 2.27% 2.5 L of Nutrineal 2.5 L of Extraneal	5 L of Physioneal 40 Glucose 1.36% 5 L of Physioneal 40 Glucose 2.27% 2.5 L of Nutrineal 2.5 L of Extraneal
Treatment	Hydrochlorothiazide suspended Cinacalcet 60 mg daily Sevelamer 9.6 gr daily	Cinacalcet 60 daily Sevelamer 9.6 gr daily	Cinacalcet 90 daily Sevelamer 9.6 gr daily	Cinacalcet 90 mg/4 days a week Cinacalcet 120 mg/3 days a week Sevelamer 9.6 gr daily	Cinacalcet 90 mg/5 days a week Cinacalcet 120 mg/ days a week Sevelamer 9.6 gr daily
sCa (mg/dL)	9.9	10	9.2	9.5	8.6
sP (mg/dL)	8.8	7.6	5.4	7	5.6
Ca × P (mg^2^/dL^2^)	87.12	76	49.68	66.5	48.16
Alkaline phosphatase (U/L)	68	73	117	123	125
25-hydroxy vitamin D (ng/mL)	10.5	9.4	11.5	13.1	----
PTH (pg/mL)	901	578	440	605	408

APD, ambulatory peritoneal dialysis; CAPD, continuous ambulatory peritoneal dialysis; CCr, creatinine clearance; D/P Cr, dialysate-to-plasma ratio of creatinine; sCa, serum calcium; sP, serum phosphorus; PET, peritoneal equilibration test; PTH, parathyroid hormone.

## Data Availability

No new data were created or analyzed in this study. The data used to support the findings of this study are available from the corresponding author on request (contact J.C.D.L.F., jflomer@mde.es).
